# Supplementary material for: Preparing GIS data for analysis of stream monitoring data: The R package openSTARS
Source: PLoS One. 2020 Sep 17;15(9):e0239237. doi: 10.1371/journal.pone.0239237 (PMC7498020; doi:10.1371/journal.pone.0239237)
Supplement: S1 File — (PDF) [file pone.0239237.s001.pdf]

Supporting Information for the manuscript **Preparing GIS data for analysis of stream monitoring data: The R package openSTARS**

Mira Kattwinkel, Eduard Szöcs, Erin Peterson, Ralf B. Schäfer

S1 A Bland-Altman plot comparing catchment area and area of arable land for STARS and openSTARS

Similar to Fig 3 in the main text, the Bland-Altman plot indicates that there is no systematic difference between the two tools in calculating catchment attributes in the case study (S1 Fig). The plot shows the difference in the attributes calculated with STARS and openSTARS, respectively, on the y-axis, and the mean of the value pairs on the x-axis. For the catchment area of sampling sites, one outlier can be found (S1 Fig A; the same as marked in Fig 2 in the main text) and two for the area of arable land within the catchments (S1 Fig B). The reasons for these outliers are discussed below.

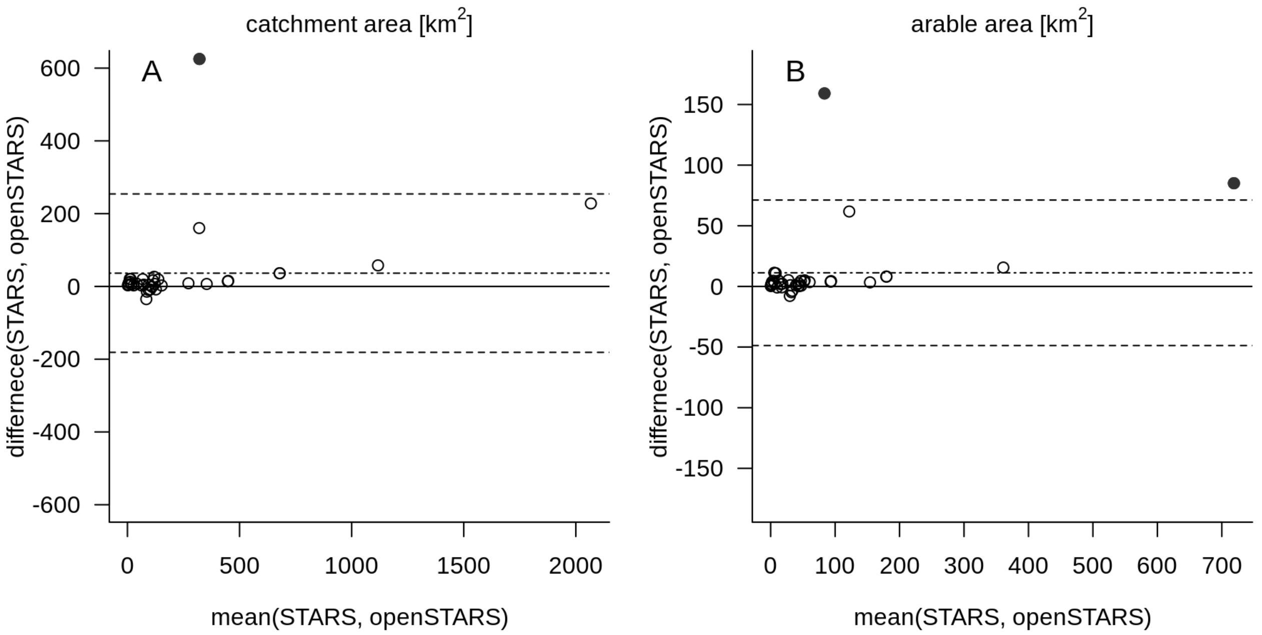

S1 Fig: Bland-Altman plot comparing catchment attributes for the sampling sites calculated with STARS and openSTARS. A: catchment area; B: arable land use within the catchment. Solid line indicates zero difference between the two tool, the dash-dotted line gives the mean difference and the dashed lines indicate the mean difference+/- 1.96 standard derivations of the difference; outliers are marked with solid black dots.

S1 B Differences in catchment areas for STARS and openSTARS in the case study

In the case study presented, there is one site where the STARS toolbox yield a much larger catchment area compared to openSTARS (Fig 3A), and tow sites where the calculated area of arable land within the catchment is also much larger. For one site (left in Fig 3) this can be attributed to the fact that the site is snapped to a smaller tributary created in openSTARS, which is lacking in the streams dataset used in STARS (S2 Fig). For the other site, the stream network is discontinued at one point in openSTARS resulting in a smaller catchment (approx 1950 km^2 compared to 2180 km^2) and a particularly smaller area of arable land (S3 Fig).

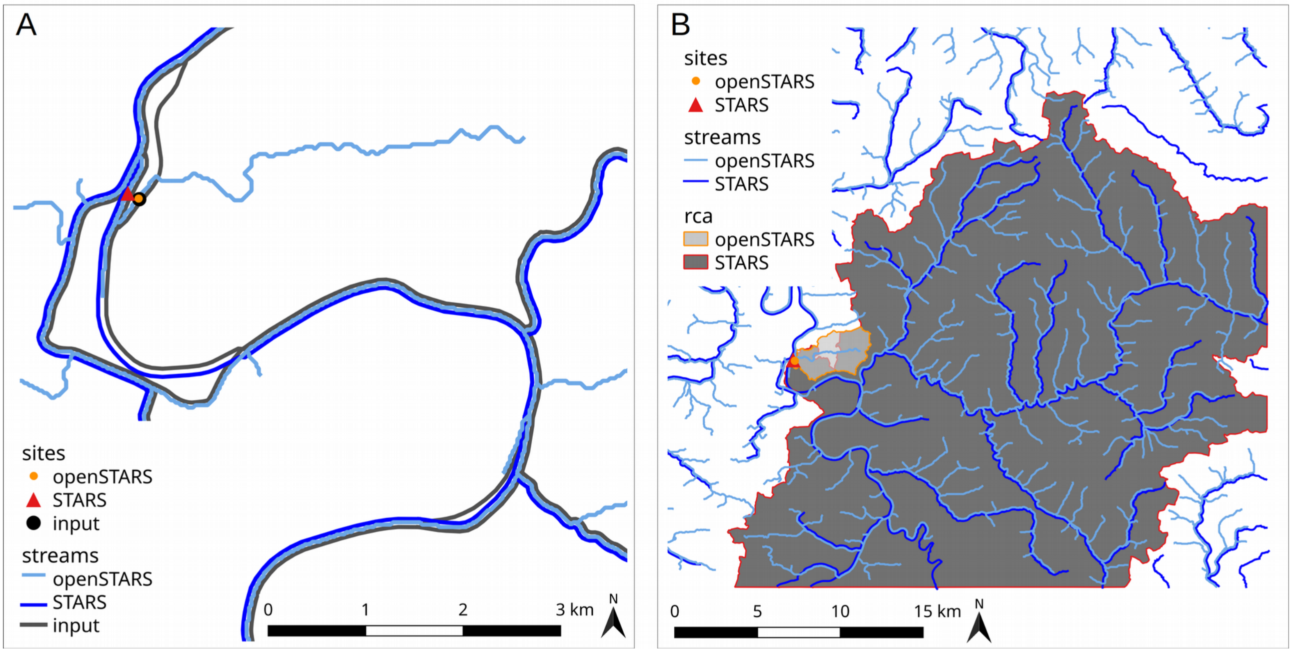

S2 Fig: Comparison of openSTARS and STARS for the left marked outlier in Figure 3 in the main text. Streams for STARS and openSTARS with slight offset compared to the input data. A: Stream courses and site positions. For openSTARS the site is snapped to a small tributary resulting in a far smaller catchment. B: Reach contributing areas (rca).

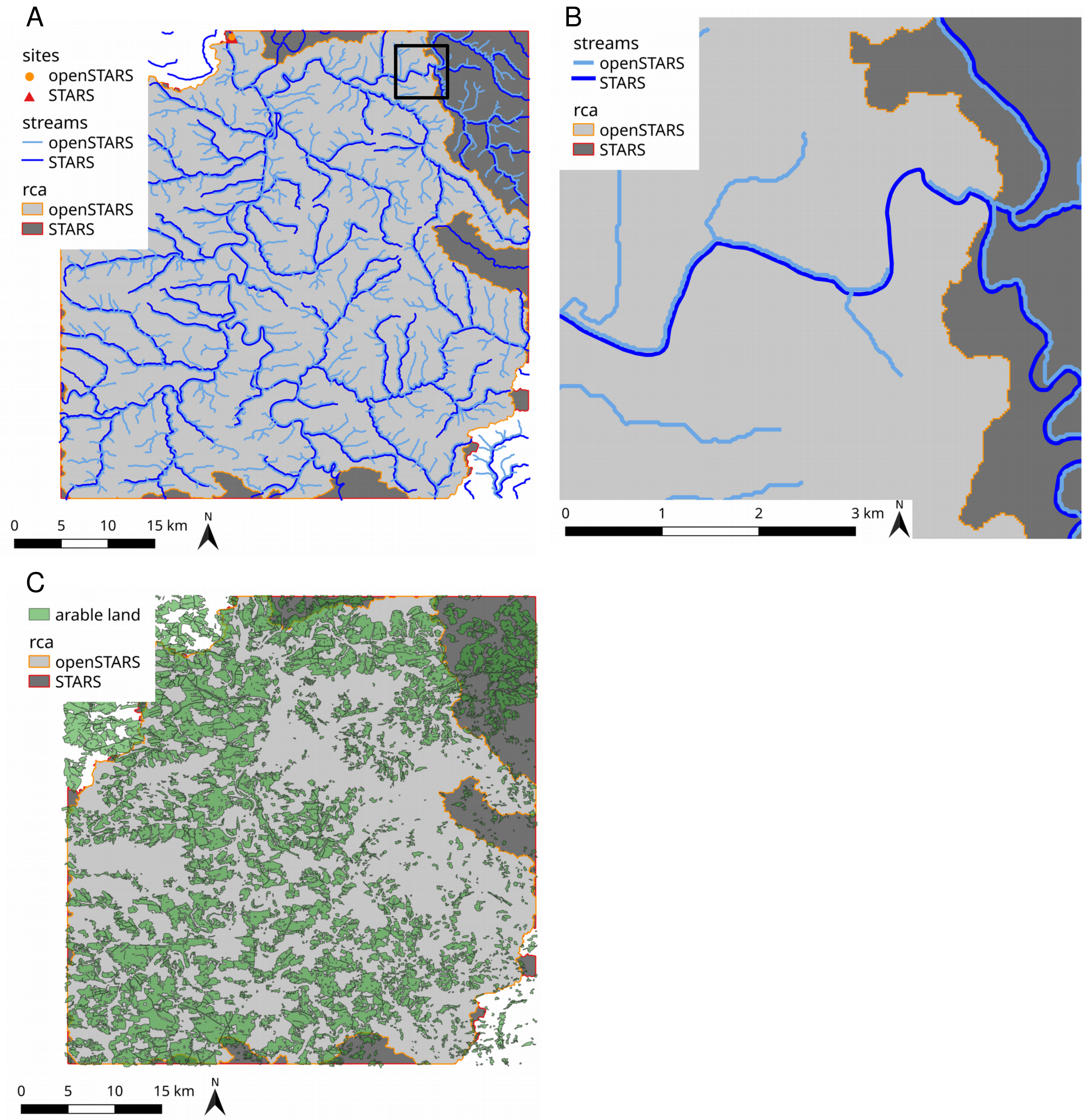

S3 Fig: Comparison of openSTARS and STARS for the right marked outlier in Figure 3 in the main text. Streams for STARS with slight offset. A: Stream courses, site positions and reach contributing areas (rca). For openSTARS the rca is a bit smaller because one stream consists of two different parts compared to STARS. The black square marks the area zoomed in B. B: Zoom to the the respective region. Note the separation in the light blue streams constructed with openSTARS. C: Distribution of arable land. The relatively high proportion in the North-Eastern corner results in the larger area of arable land in the catchment for this site derived with STARS.
