# Supplementary material for: Preparing GIS data for analysis of stream monitoring data: The R package openSTARS
Source: PLoS One. 2020 Sep 17;15(9):e0239237. doi: 10.1371/journal.pone.0239237 (PMC7498020; doi:10.1371/journal.pone.0239237)
Supplement: S2 File — (PDF) [file pone.0239237.s002.pdf]

Supporting Information 2

Mira Kattwinkel

May 21, 2020

Supporting Information for the manuscript 'Preparing GIS data for analysis of stream monitoring data: The R package openSTARS'

Mira Kattwinkel, Eduard Szöcs, Erin Peterson, Ralf B. Schäfer

S2 Complete openSTARS Workflow (commented R code)

Preparations

Data source

This code uses the freely available data set of North Carolina, USA (Neteler and Mitasova 2008; https://grass.osgeo.org/download/data/). The parts of the data used in this example are provided with the openSTARS package installation.

GRASS installation

GRASS GIS (version 7.0 or greater) must be installed (https://grass.osgeo.org/download/). Additionally, some GRASS extensions must be present: 'r.stream.basins', 'r.stream.distance', 'r.stream.order', and 'r.hydrodem'. They can be activated within GRASS under Settings / Addons extensions / Install extensions from addons [g.extension]. In the menu click on 'raster', select the respective extension and hit install (or double-click); close GRASS and restart R.

If you do not get a list to choose the extensions from in the GRASS GUI, open the tab 'Console' in the lower part of the Layer Manager window in GRASS; type `g.extension` in the text field and hit enter; in the opening window, type the name of the extension to be installed, choose 'add' as 'Operation to be performed'; click 'Run'. Repeat for all four extensions. You might get an error that metadata cannot be read, but the installation should work successfully anyway. Close the new window. To check the installation, type e.g. `r.hydrodem` into the text field and hit enter: A new window to run the tool should open; close it. Close GRASS and restart R.

Install R packages required for this example

The following packages are used in this example and must be installed and loaded. There might be a `WARNING` message about Rtools that can be ignored.

```r
# for plotting maps
if(!require("tmap")){
  install.packages("tmap")
  install.packages("tmaptools")
}
## Loading required package: tmap

library(tmap)
library(tmaptools)
```

Install and load openSTARS

Installation from CRAN repository:

```r
install.packages("openSTARS")
```

For the lastest development version of openSTARS install it from GitHub (carefull, might be experimental). There might be another `WARNING` message about Rtools that can be ignored.

```r
# to install openSTARS from github
if(!require("devtools")){
  install.packages("devtools")
  }
devtools::install_github("MiKatt/openSTARS", ref = "dev")
```

Load the package:

```r
library(openSTARS)
## Loading required package: data.table

## Loading required package: rgrass7

## Loading required package: XML

## GRASS GIS interface loaded with GRASS version: (GRASS not running)
```

Workflow

A - Load Data

Initiate and setup GRASS

First, a GRASS session must be initiated and setup. Adjust the paths to the GRASS installation (`gisBase`) and, if needed, to the GRASS data base where all GRASS files will be stored (`gisDbase`) to those on your system. The name of the GRASS location within the `gisDbase` can be given (`location`). The projection and extent of the GRASS location is based on that one of the digital elevation model (`dem`) and is used for all input and output files. On Windows systems, you might get a warinig '`WARNING: Concurrent mapset locking is not supported on Windows`' that can be ignored.

Please use data in a metric coordinate reference system (CRS) appropriate for the study region (i.e. no long/lat CRS) for all input data. Otherwise particularly the network correction on 'correct_colplex_confluences' might not work proberly. Suggestions for CRS can e.g. be found here http://epsg.io/.

```
# give paths to GRASS and where to store the GRASS data base
# Linux e.g.
grass_program_path <- "/usr/lib/grass78/"
# Windows e.g.
# grass_program_path <- "c:/Program Files/GRASS GIS 7.6"
working_dir <- file.path(Sys.getenv("HOME"), "grass_workflow")
grass_db_path <- file.path(working_dir, "gassDB")
dir.create(working_dir)
## Warning in dir.create(working_dir): '/home/mira/grass_workflow' already exists
setwd(working_dir)

# specify the path to the digital elevation model
dem_path <- system.file("extdata", "nc", "elev_ned_30m.tif", package = "openSTARS")
setup_grass_environment(dem = dem_path,
                        gisBase = grass_program_path,
                        gisDbase = grass_db_path,
                        location = "nc_openSTARS",
                        remove_GISRC = TRUE,
                        override = TRUE
                        )
```

```
gmeta()
## gisdbase    /home/mira/grass_workflow/gassDB
## location    nc_openSTARS
## mapset      PERMANENT
## rows        450
## columns     500
## north       228500
## south       215000
## west        630000
## east        645000
## nsres       30
## ewres       30
## projection  +proj=lcc +lat_0=33.75 +lon_0=-79 +lat_1=36.1666666666667
## +lat_2=34.3333333333333 +x_0=609601.22 +y_0=0 +no_defs +a=6378137
## +rf=298.257222101 +towgs84=0.000,0.000,0.000 +type=crs +to_meter=1
```

Load data into the GRASS location

Then, `import_data` imports all data into GRASS (DEM, sampling sites and other optional data). Optional data includes a stream network to burn into the DEM (see `derive_streams`), prediction sites if they have already been created in a different program (prediction sites can be created using `calc_prediction_sites` at a later stage), and raster and vector maps of potential predictor variables that will be intersected with the catchments of the sites (see `calc_attributes_edges` and `calc_attributes_sites_approx`, or `calc_attributes_sites_exact`).

The DEM is loaded into the GRASS database as a raster map named `dem`, the sites as a vector map named `sites_o` and the (optional) stream network as a vector map named `streams_o`. Prediction sites are stored

under their base file name, potential predictors either using their base file names or the ones provided in `predictor_r_names` and `predictor_v_names`, respectively.

Note that vector data is reprojected on the fly in this import, while raster data is not reprojected as it should be done manually (due to the changes this might cause to the resolution etc.). It can be checked before the import if the current region (as defined by the DEM) and other raster maps have the same projection. If this is not the case, they should be reprojected before, so that the dem and all raster files are in identical CRS'. Please note that providing all data including vector data in the same CRS is best to avoid errors during import.

```
preds_r_path <- system.file("extdata", "nc", "landuse_r.tif", package = "openSTARS")
check_projection(preds_r_path)
## current location

## [1] "PROJCS[\"unknown\","
## [2] " GEOGCS[\"grs80\","
## [3] " DATUM[\"North_American_Datum_1983\","
## [4] " SPHEROID[\"Geodetic_Reference_System_1980\",6378137,298.257222101],"
## [5] " AUTHORITY[\"EPSG\",\"6269\"]],"
## [6] " PRIMEM[\"Greenwich\",0,"
## [7] " AUTHORITY[\"EPSG\",\"8901\"]],"
## [8] " UNIT[\"degree\",0.0174532925199433,"
## [9] " AUTHORITY[\"EPSG\",\"9122\"]]],"
## [10] " PROJECTION[\"Lambert_Conformal_Conic_2SP\"],"
## [11] " PARAMETER[\"latitude_of_origin\",33.75],"
## [12] " PARAMETER[\"central_meridian\",-79],"
## [13] " PARAMETER[\"standard_parallel_1\",36.1666666666667],"
## [14] " PARAMETER[\"standard_parallel_2\",34.3333333333333],"
## [15] " PARAMETER[\"false_easting\",609601.22],"
## [16] " PARAMETER[\"false_northing\",0],"
## [17] " UNIT[\"metre\",1,"
## [18] " AUTHORITY[\"EPSG\",\"9001\"]],"
## [19] " AXIS[\"Easting\",EAST],"
## [20] " AXIS[\"Northing\",NORTH]]"

## landuse_r.tif

## Projection seems to match current location

## [1] "PROJCS[\"unknown\","
## [2] " GEOGCS[\"grs80\","
## [3] " DATUM[\"North_American_Datum_1983\","
## [4] " SPHEROID[\"Geodetic_Reference_System_1980\",6378137,298.257222101],"
## [5] " AUTHORITY[\"EPSG\",\"6269\"]],"
## [6] " PRIMEM[\"Greenwich\",0,"
## [7] " AUTHORITY[\"EPSG\",\"8901\"]],"
## [8] " UNIT[\"degree\",0.0174532925199433,"
## [9] " AUTHORITY[\"EPSG\",\"9122\"]]],"
## [10] " PROJECTION[\"Lambert_Conformal_Conic_2SP\"],"
## [11] " PARAMETER[\"latitude_of_origin\",33.75],"
## [12] " PARAMETER[\"central_meridian\",-79],"
## [13] " PARAMETER[\"standard_parallel_1\",36.1666666666667],"
## [14] " PARAMETER[\"standard_parallel_2\",34.3333333333333],"
## [15] " PARAMETER[\"false_easting\",609601.22],"
```

```
## [16] " PARAMETER[\"false_northing\",0],"
## [17] " UNIT[\"metre\",1,"
## [18] " AUTHORITY[\"EPSG\",\"9001\"]],"
## [19] " AXIS[\"Easting\",EAST],"
## [20] " AXIS[\"Northing\",NORTH]]"
```

```
# give file paths to
# sampling sites
sites_path <- system.file("extdata", "nc", "sites_nc.shp", package = "openSTARS")
# potential predictors in raster format
preds_r_path <- system.file("extdata", "nc", "landuse_r.tif", package = "openSTARS")
# potential predictors in vector format
preds_v_path <- c(system.file("extdata", "nc", "geology.shp", package = "openSTARS"),
                  system.file("extdata", "nc", "pointsources.shp", package = "openSTARS"))
# existing stream network
streams_path <- system.file("extdata", "nc", "streams.shp", package = "openSTARS")

import_data(dem = dem_path, sites = sites_path, streams = streams_path,
            predictor_vector = preds_v_path, predictor_v_names = c("geology", "psources"),
            predictor_raster = preds_r_path)
## Loading DEM into GRASS as 'dem' ...

## Loading sites into GRASS as 'sites_o' ...

## Loading raster predictor variables into GRASS as 'landuse_r' ...

## Loading vector predictor variables into GRASS as 'geology', 'psources' ...

## Loading streams into GRASS as 'streams_o'  ...
```

The data is plotted below. Vector data is read into R from the GRASS location with `readVECT`, raster data with `readRAST`. Please note that here and below there might be warning messages `Discarded datum unknown in CRS definition, but +towgs84= values preserved` or `CRS object has comment, which is lost in output`; they can be ignored here as they are not relevant for plotting.

```
# read in data
dem <- readRAST("dem", ignore.stderr = TRUE)
sites_o <- readVECT("sites_o", ignore.stderr = TRUE)
streams_o <- readVECT("streams_o", ignore.stderr = TRUE)
psources <- readVECT("psources", ignore.stderr = TRUE)
geology <- readVECT("geology", ignore.stderr = TRUE)
landuse <- readRAST("landuse_r", ignore.stderr = TRUE)

# create dummy columns to get a legend in tmap (tm_symbols, tm_lines) with just one color
sites_o$col <- 1
streams_o$col <- 1
psources$col <- 1
```

```
# dem, original streams and sites (streams_o and sites_o)
tmap_mode("plot")
## tmap mode set to plotting

tm_shape(dem) +
```

```r
  tm_raster("dem", palette = terrain.colors(n = 9), n = 9,
            title = "digital elevation \nmodel [m asl]", style = "cont") +
  tm_shape(streams_o) +
  tm_lines(col = "col", lwd = 1.5, legend.col.show = TRUE, palette = "blue4",
           title.col = "", legend.format = list(fun=function(x) "input streams")) +
  tm_shape(sites_o) +
  tm_symbols(col = "col", palette = "red", size = 0.4,
             legend.col.show = TRUE, title.col = "",
             legend.format = list(fun=function(x) "input sampling sites")) +
  #tm_view(symbol.size.fixed    = TRUE) +  # helpful for tmap_mode('view')
  tm_layout(scale = 1, legend.bg.color = T, legend.position = c("left", "bottom"))
```

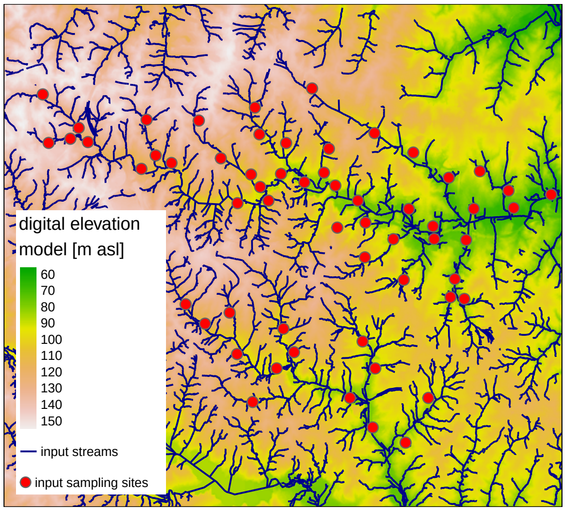


```r
# geology
tm_shape(geology) +
  tm_polygons(col = "GEO_NAME", palette = RColorBrewer::brewer.pal(8, "Accent"),
              title = "geology", n = 8)  +
  tm_layout(scale = 1, legend.bg.color = T, legend.position = c("left", "bottom"))
```

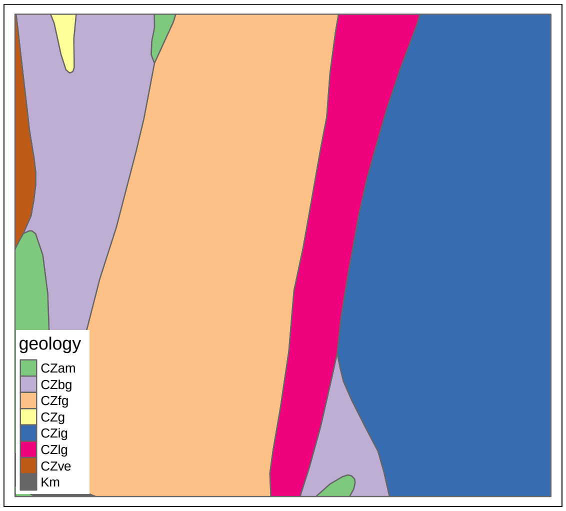

```r
# landuse and point sources
tm_shape(landuse) +
  tm_raster(style = "cat", title = "land use",
            palette = c("red", "goldenrod", "green", "forestgreen",
                        "darkgreen", "blue", "lightblue"),
            labels = c("developed", "agriculture", "herbaceous", "shrubland",
                       "forest", "water", "sediment")) +
  tm_shape(psources) +
  tm_symbols(col = "col", palette = "black",  border.col = "white", border.lwd = 2,
             size = 0.6, legend.col.show = TRUE,
             title.col = "", legend.format = list(fun=function(x) "point sources")) +
  tm_layout(scale = 1, legend.bg.color = T, legend.position = c("left", "bottom"))
```

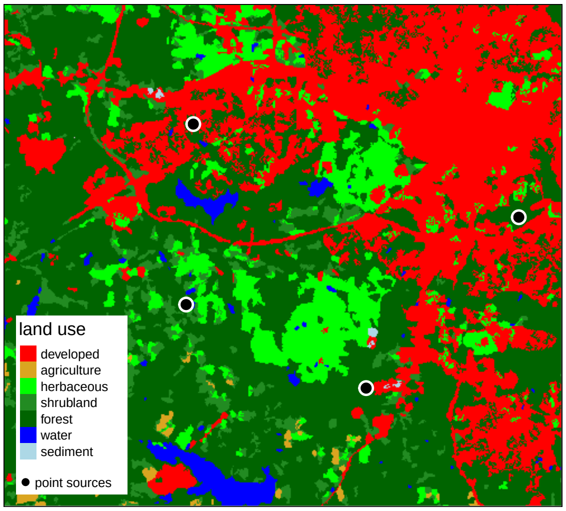

B - Derive and clean stream network

Derive streams from DEM

Next, the streams must be derived from the DEM (saved as `streams_v` in the GRASS data base). An existing stream network (if provided to `import_data` before) can be burnt into the DEM to force the streams derived from the DEM to mapped ones. It is not possible to use a given stream network directly because it might lack topological information needed in the consecutive steps.

Additional specifications on the stream derivation can be provided (see `?derive_streams` and the GRASS function r.stream.extract for details). E.g., `accum_threshold` (default `= 700`) gives the accumulation threshold, i.e. the minimum number of raster cells of the DEM that initiate a new stream; smaller values result in more small tributaries. `condition` (default `= TRUE`) determines if the DEM is conditioned before further processing, i.e. if sinks are removed. It is recommended to use it but it might take some time.

```r
# With burn-in and changes to the default parameter settings;
derive_streams(accum_threshold = 100, condition = TRUE, clean = TRUE, burn = 10)
## Conditioning DEM ...

## Burning streams into DEM ...

## Deriving streams from DEM ...
```

```
## Calculating stream topology ...

## Derived streams saved as 'streams_v'.
```

```
# read in the derived stream network
streams <- readVECT("streams_v", ignore.stderr = TRUE)
# create dummy columns to get a legend in tmap (tm_symbols, tm_lines) with just one color
streams$col <- 1

# plot
tm_shape(dem) +
  tm_raster("dem", palette = terrain.colors(n = 9), n = 9,
            title = "digital elevation \nmodel [m asl]", style = "cont") +
  tm_shape(streams_o) +
  tm_lines(col = "col", lwd = 1, legend.col.show = TRUE, palette = "blue4",
           title.col = "", legend.format = list(fun=function(x) "input streams")) +
  tm_shape(streams) +
  tm_lines(col = "col", palette = "dodgerblue", legend.col.show = TRUE, lwd = 2,
           title.col = "", legend.format = list(fun=function(x) "derived streams")) +
  tm_layout(scale = 1, legend.bg.color = T, legend.position = c("left", "bottom"))
```

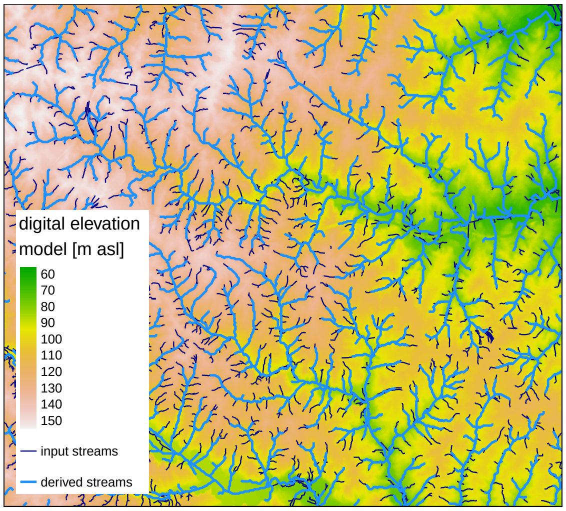

Check and correct the network

The GRASS functions used in `derive_streams()` may results in stream confluences of more than three segments, i.e. where more than two line segments flow into a node. These parts must be corrected before further processing. Other topological errors as mentioned for the ArcGIS toolbox STARS do not occur if the stream network is derived from a DEM.

```
cp <- check_compl_confluences()
## There are complex confluences in the stream network. Please run
correct_compl_confluences() for correction.

if (cp)
  correct_compl_confluences()
```

```
## Fixing 6 complex confluences with 3 upstream segments each

## Original stream topology file moved to 'streams_v_o3'.

## Breaking lines and moving vertices ...

## Updating topology ...

## Complex confluences were removed. Please check changed features in
'streams_v'.
```

```
# plot
streams <- readVECT('streams_v', ignore.stderr = TRUE)
streams_orig <- readVECT('streams_v_o3', ignore.stderr = TRUE)
streams$col <- 1
streams_orig$col <- 1
tmap_mode("plot")
## tmap mode set to plotting


# bounding box to zoom to the relevant part
bb <- cbind(c(640100, 219740), c(640150, 219790))
tm_shape(streams_orig, bbox = bb, unit = "m") +
  tm_lines(col = "col", lwd = 2, legend.col.show = TRUE, palette = "dodgerblue",
           title.col = "", legend.format = list(fun=function(x) "original streams")) +
  tm_shape(streams) +
  tm_lines(col = "col", lwd = 3, lty = 2, palette = "darkblue", legend.col.show = TRUE,
           title.col = "", legend.format = list(fun=function(x) "corrected streams")) +
  tm_scale_bar()
```

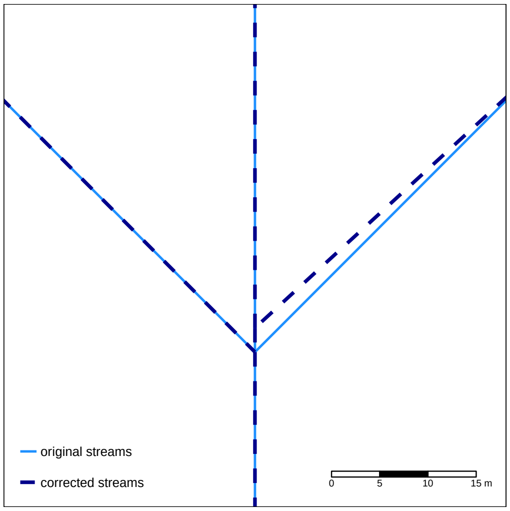

To mark the changed segments in red and use interactive zooming, use `tmap_mode("view")` (it is not possible to show the output in a pdf):

```
# create a new column named 'changed.str' with 'unchanged' / 'changed' for a nice legend
streams@data$changed.str <- ifelse(streams@data$changed == 1, "changed", "unchanged")
# use tmap_mode("view") to enable interactive zooming
```

```r
tmap_mode("view")
tm_shape(streams) +
  tm_lines(col = "changed.str", lwd = 2, legend.col.show = TRUE,
           palette = c("red", "grey"),
           title.col = "changed segments") +
  tm_shape(streams_orig) +
  tm_lines(col = "col", lwd = 1, lty = 2, legend.col.show = TRUE, palette = "dodgerblue",
           title.col = "", legend.format = list(fun=function(x) "before correction")) +
  tm_basemap(NULL) +
  tm_layout(scale = 1, legend.bg.color = T) +
  tm_scale_bar()
```

Delete lakes

Optionally, lakes can be deleted from the network. This might be necessary because when the stream network is derived from a dem, the streams will just cross lakes or ponds. However, the flow is disconnected here and the relationship between sampling points upstream and downstream of a lake is not clear. This function intersects the stream network with a given vector map of lakes, deletes the stream segments in the lake, breaks those that cross its borders and assigns a new, updated topology.

```r
lakes_path <- system.file("extdata", "nc", "lakes.shp", package = "openSTARS")
delete_lakes(lakes = lakes_path)
## Importing lakes ...

## Deleting stream segments intersecting with lakes ...

## Updating topology ...

## Updating out_dist ...
```

```r
# plot
streams <- readVECT('streams_v', ignore.stderr = TRUE)
streams_with_lakes <- readVECT('streams_v_prev_lakes', ignore.stderr = TRUE)
lakes <- readVECT('lakes', ignore.stderr = TRUE)

streams$col <- 1
streams_with_lakes$col <- 1
lakes$col <- 1

tmap_mode("plot")
## tmap mode set to plotting

tm_shape(lakes, bbox = streams ) +
  tm_polygons(col = "col", palette = "grey",
              title = "", n = 1, legend.format = list(fun=function(x) "lakes")) +
  tm_shape(streams_with_lakes) +
  tm_lines(col = "col", lwd = 4, legend.col.show = TRUE, palette = "dodgerblue",
           title.col = "", legend.format = list(fun=function(x) "original streams")) +
  tm_shape(streams) +
  tm_lines(col = "col", lwd = 1, lty = 1, palette = "darkblue", legend.col.show = TRUE,
           title.col = "", legend.format = list(fun=function(x) "streams, lakes deleted")) +
  tm_scale_bar() +
  tm_layout(scale = 1, legend.bg.color = T, legend.position = c("left", "bottom"))
```

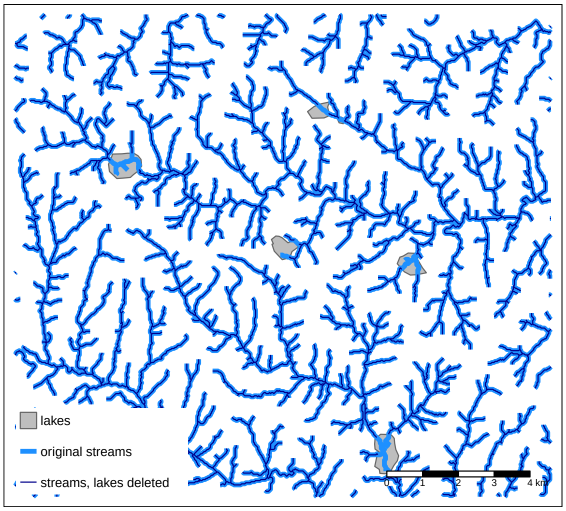

Prepare edges

Now, information needed for the SSN object are derived for the streams and stored in a new vector map `edges` based on `streams_v` created with `derive_streams()`.

```
calc_edges()
## Calculating reach contributing area (RCA) ...

## Calculating upstream catchment areas ...


edges <- readVECT("edges", ignore.stderr = TRUE)
head(edges@data, n = 4)
##   cat stream prev_str01 prev_str02 next_str flow_accu changed netID rid
## 1   1      1          0          0       67  147.6439       0    21   0
## 2   2      2          0          0       67  363.1734       0    21   1
## 3   3      3          0          0      216  914.0473       0    16   2
## 4   4      4          0          0       68  238.7341       0    18   3
##   OBJECTID  Length  upDist H2OArea rcaArea
## 1        1  434.56 2712.31  0.2511  0.2511
## 2        2  554.56 2832.31  0.3312  0.3312
## 3        3 1059.41 2248.23  0.6093  0.6093
## 4        4  501.84 3247.65  0.2205  0.2205
```

`edges` now holds the derived network plus attributes needed for the .ssn object:

- network identifier (netID)
- reach identifier (rid)
- OBJECTID (= stream, to conform ArcGIS data standards)
- stream segment length (Length)
- upstream distance, i.e. distance from the outlet of the network to the start (upstream node) of the stream segment (upDist)
- total catchment area (H2OArea)

- reach contributing area (rcaArea)

The additional fields hold information about the network: 'next_str' is the 'stream' this segment flows into, 'prev_str01' and 'prev_str02' are the two segments that flow into this segment.

C - Prepare sampling and prediction sites

Prepare sites

Similar, the necassary information are derived for the sites. Additionally, sampling sites often do not lay exactly on the stream network (due to GPS imprecision, stream representation as lines, derivation of streams from a DEM, etc.). To assign an exact position of the sites on the network they are moved to the closest stream segment (snapped). Optionally, a maximum snapping distance can be provided (`max_dist`); sites further away from any edge segment will be deleted. If the reported snapping distance is too large, deriving a finer stream network might be an option (smaller `accum_threshold` in `derive_streams`).

```
calc_sites()
## Preparing sites 'sites' ...

## Snapping sites to streams ...

## Warning in proj4string(sites): CRS object has comment, which is lost in output
## Maximum snapping distance found: 209.2 m

## Setting pid and locID ...

## Assigning netID and rid ...

## Calculating upDist ...


sites <- readVECT("sites", ignore.stderr = TRUE)
head(sites@data, n = 4)
##   cat site_id value str_edge     dist   NEAR_X   NEAR_Y locID pid netID rid
## 1   1       1     1       65 85.04240 631046.1 226074.1     1   1    21  64
## 2   2       4     1      174 47.80371 632011.9 225175.7     2   2    21 171
## 3   3       5     1       67 29.83919 631203.4 224771.5     3   3    21  66
## 4   4       7     1      175 31.22663 631787.3 224883.8     4   4    21 172
##    upDist distalong     ratio
## 1 2828.29  147.9922 0.1536049
## 2 1368.34  114.8528 0.7124438
## 3 2012.90  264.8528 0.4950953
## 4 1286.33  104.7310 0.6591677
```

`sites` now represents the snapped sampling sites and attributes needed for the .ssn object:

- point identifier (pid), unique to each measurement
- location identifier (locID), unique to each location
- network identifier (netID)
- reach identifier of the edge segment the point lies on (rid)
- upstream distance (upDist), i.e. the distance from each site to the network outlet calculated using r.stream.distance.

- distance ratio (ratio), i.e. the ratio: (distance from the downstream node of the edge to the site location)/(total length of the edge the site resides on).

Additional fields hold information on the snapping: distance of the original site to the closest edge ('dist'), i.e. how far the point was moved, and the new x and y coordinates ('NEAR_X', 'NEAR_Y'). The filed 'stream_edge' gives the 'stream' of the stream segment the point lies on. It is used to identify the edge the point lies on to extract the 'rid'.

Plot the original and snapped sites:

```
# create dummy columns to get a legend in tmap (tm_symbols, tm_lines) with just one color
sites$col <- 1
edges$col <- 1

tmap_mode("plot")
## tmap mode set to plotting

tm_shape(edges) +
  tm_lines(col = "col", palette = "darkblue", legend.col.show = TRUE,
           title.col = "", legend.format = list(fun=function(x) "edges")) +
  tm_shape(sites_o) +
  tm_symbols(col = "col", palette = "grey35", size = 0.4, legend.col.show = TRUE,
             legend.format = list(fun=function(x) "input sampling sites"),
             title.col = "") +
  tm_shape(sites) +
  tm_symbols(col = "col", palette = "red", size = 0.2, legend.col.show = TRUE,
             title.col = "", legend.format = list(fun=function(x) "snapped sites")) +
  tm_layout(scale = 1, legend.bg.color = T, legend.position = c("left", "bottom"))
```

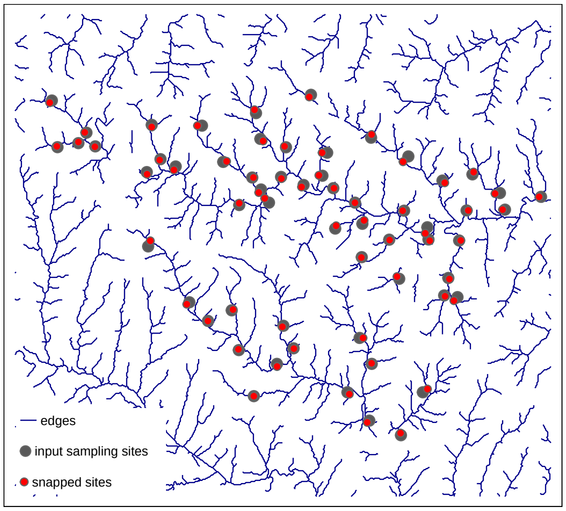

For an interactive map use `tmap_mode("view")`:

```
tmap_mode("view")
tm_shape(edges) +
  tm_lines(col = "col", palette = "darkblue", legend.col.show = TRUE,
           title.col = "", legend.format = list(fun=function(x) "edges")) +
  tm_shape(sites_o) +
  tm_symbols(col = "col", palette = "grey35", size = 0.08, legend.col.show = TRUE,
```

```r
            title.col = "", legend.format = list(fun=function(x) "input sampling sites")) +
  tm_shape(sites) +
  tm_symbols(col = "col", palette = "red", size = 0.04, legend.col.show = TRUE,
            legend.format = list(fun=function(x) "snapped sites"),
            title.col = "") +
  tm_basemap(NULL) +
  tm_layout(scale = 1, legend.bg.color = T) +
  tm_scale_bar() +
  tm_view(symbol.size.fixed = T)
```

If predictions sites were created before in a different program and have been imported by 'import_data' they should be treated similarly:

```r
calc_sites(predictions = prediction_sites_name)
```

Prepare prediction sites

Prediction sites can be created along the streams. Either the distance between the sites must be provided (`dist`) or the approximate number of sites that shall be created (`nsites`). Additionally, the creation can be restricted to certain networks (`netIDs`). The sites will be assigned regularly on the stream network, starting from the outlets. Similar as for the sampling sites, attributes needed for .ssn object are assigned:

- point identifier (pid), unique
- location identifier (locID), unique to each location
- network identifier (netID)
- reach identifier of the edge segment the point lies on (rid)
- upstream distance (upDist), i.e. the distance from each site to the network outlet calculated using r.stream.distance.
- distance ratio (ratio), i.e. the ratio: (distance from the downstream node of the edge to the site location)/(total length of the edge the site resides on).

```r
calc_prediction_sites(predictions = "preds", nsites = 200, netIDs = 60)
## Calculating point positions ...

## Creating attribute table ...

## Setting cat_edge ...

## Setting pid and locID ...

## Assigning netID and rid ...

## Calculating upDist ...

## Calculating distance ratio ...
```

Inspect and plot the created prediction sites:

```r
# inspect and plot
pred_sites <- readVECT("preds", ignore.stderr = TRUE)
head(pred_sites@data, n = 4)
```

```
##   cat str_edge         dist pid rid distalong     ratio locID netID   upDist
## 1   1        8 2.910383e-11   1   7        79 0.4977111     1    60 16050.25
## 2   2       14 2.910383e-11   2  13       139 0.5202099     2    60 18618.19
## 3   3       18 0.000000e+00   3  17       278 0.2577958     3    60 17976.47
## 4   4       24 0.000000e+00   4  23       266 0.2654572     4    60 17976.04
##     NEAR_X   NEAR_Y
## 1 635209.6 226170.4
## 2 633469.1 226029.1
## 3 633405.0 223283.3
## 4 633308.9 223355.0

pred_sites$col <- 1

tmap_mode("plot")
## tmap mode set to plotting

tm_shape(edges) +
  tm_lines(col = "col", palette = "darkblue", legend.col.show = TRUE,
           title.col = "", legend.format = list(fun=function(x) "edges")) +
  tm_shape(pred_sites) +
  tm_symbols(col = "col", palette = "orange", size = 0.2, legend.col.show = TRUE,
             title.col = "", legend.format = list(fun=function(x) "prediction sites")) +
  tm_layout(scale = 1, legend.bg.color = T, legend.position = c("left", "bottom"))
```

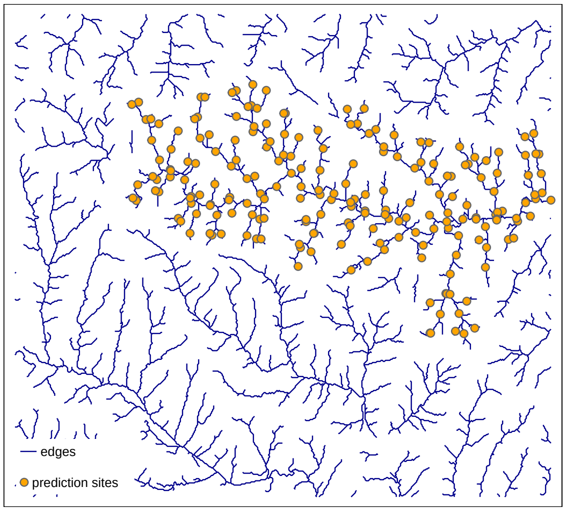

Restrict the network

Optionally, the stream network (`edges`) can be restricted to certain netIDs, or to the parts with sampling and prediction sites. This may ease the computational burden when intersecting edges with potential predictors and also decreases the data to be saved in the SSN object.

```
restrict_network(sites = "sites", keep = TRUE, filename = "edges_o")
## Original edges moved to edges_o.

## Deleting edges with netIDs other than 21, 60, 12, 44, 53, 54 ...
```

```r
# inspect and plot
edges_new <- readVECT("edges", ignore.stderr = TRUE)
edges_old <- readVECT("edges_o", ignore.stderr = TRUE)
edges_new$col <- 1
edges_old$col <- 1

tmap_mode("plot")
## tmap mode set to plotting

tm_shape(edges_old) +
  tm_lines(col = "col", palette = "dodgerblue", legend.col.show = TRUE,
           title.col = "", legend.format = list(fun=function(x) "before restriction")) +
  tm_shape(edges_new) +
  tm_lines(col = "col", lwd = 1, lty = 2, legend.col.show = TRUE, palette = "darkblue",
           title.col = "", legend.format = list(fun=function(x) "after restriction")) +
  tm_shape(sites) +
  tm_symbols(col = "col", palette = "red", size = 0.2, legend.col.show = TRUE,
             title.col = "", legend.format = list(fun=function(x) "sampling sites")) +
  tm_layout(scale = 1, legend.bg.color = T, legend.position = c("left", "bottom"))
```

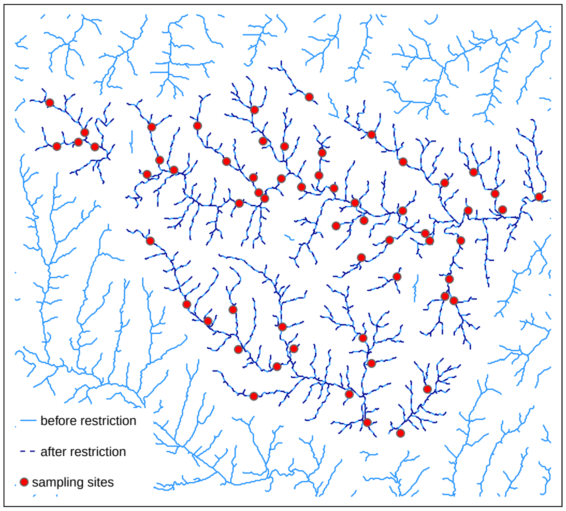

D - Derive potential predictors

Calculate attributes from raster and vector maps

Attributes (i.e. predictor variables for the regression model) can be calculated for sampling and prediction sites. There are two ways to calculates attributes:

1. approximately as described in Peterson & Ver Hoef, 2014: STARS: An ARCGIS Toolset Used to Calculate the Spatial Information Needed to Fit Spatial Statistical Models to Stream Network Data. J. Stat. Softw., 56 (2).
2. exactly by intersecting the catchment of each site (= points) with raster or vector maps;

For the approximate calculation, first attributes must be intersected with the catchments of the stream segments and then they are assigned to each site based on the distance ratio of each site. Note that the catchment area 'H2OArea' for each stream segment is calculated automatically in `calc_edges`.

Approximate calculation: calculate approximate catchment area, share of different landuse classes and geology type for the catchment of each site. The `input_attr_name` must be the same as the respective column names in edges. Optional new names for the calculated statistics can be given as `output_attr_name` (here 'A' at the end for 'approximate').

```
# first calculate attributes for edge segments
# this might take a while
calc_attributes_edges(input_raster = "landuse_r", stat_rast = "percent",
                      attr_name_rast = "luse",
                      input_vector = c("geology", "psources"),
                      stat_vect = c("percent", "count"),
                      attr_name_vect = c("GEO_NAME", "nps"), round_dig = 4)
## Intersecting raster maps ...

## Joining table raster attributes ...

## Intersecting vector maps ...

## Joining table vector attributes ...

##
## New attributes values are stored as 'lusep_1', 'lusep_2', 'lusep_3',
'lusep_4', 'lusep_5', 'lusep_6', 'lusep_7', 'CZamp', 'CZbgp', 'CZfgp', 'CZgp',
'CZigp', 'CZlgp', 'CZvep', 'Kmp', 'npsc' in 'edges'.


# then calculate approximates values for the sites
calc_attributes_sites_approx(sites_map = "sites",
                             input_attr_name = c('lusep_1', 'lusep_2', 'lusep_3',
                                                 'lusep_4', 'lusep_5', 'lusep_6',
                                                 'lusep_7', 'CZamp', 'CZbgp', 'CZfgp',
                                                 'CZgp', 'CZigp', 'CZlgp', 'CZvep',
                                                 'Kmp', 'npsc'),
                             output_attr_name = paste0(c('lusep_1', 'lusep_2', 'lusep_3',
                                                         'lusep_4', 'lusep_5', 'lusep_6',
                                                         'lusep_7', 'CZamp', 'CZbgp',
                                                         'CZfgp', 'CZgp', 'CZigp', 'CZlgp',
                                                         'CZvep', 'Kmp', 'npsc'), "A"),
                             stat = c(rep("percent",15), "count"))
##
## New attributes values are stored as 'H2OAreaA', 'lusep_1A', 'lusep_2A',
'lusep_3A', 'lusep_4A', 'lusep_5A', 'lusep_6A', 'lusep_7A', 'CZampA', 'CZbgpA',
'CZfgpA', 'CZgpA', 'CZigpA', 'CZlgpA', 'CZvepA', 'KmpA', 'npscA' in 'sites'.

sites <- readVECT("sites", ignore.stderr = TRUE)
head(sites@data, n = 4)
##   cat site_id value str_edge     dist   NEAR_X   NEAR_Y locID pid netID rid
## 1   1       1     1       65 85.04240 631046.1 226074.1     1   1    21  64
## 2   2       4     1      174 47.80371 632011.9 225175.7     2   2    21 171
## 3   3       5     1       67 29.83919 631203.4 224771.5     3   3    21  66
## 4   4       7     1      175 31.22663 631787.3 224883.8     4   4    21 172
##    upDist distalong     ratio H2OAreaA  lusep_1A lusep_2A   lusep_3A
## 1 2828.29  147.9922 0.1536049     0.61 0.3613933        0 0.00000000
## 2 1368.34  114.8528 0.7124438     2.06 0.1688907        0 0.05838198
```

```
## 3 2012.90  264.8528 0.4950953     0.73 0.5667011        0 0.09617373
## 4 1286.33  104.7310 0.6591677     1.28 0.4892905        0 0.06224900
##      lusep_4A  lusep_5A lusep_6A lusep_7A CZampA CZbgpA CZfgpA CZgpA CZigpA
## 1 0.071117562 0.5674891        0        0      0 0.9895      0     0      0
## 2 0.044203503 0.7285238        0        0      0 0.9970      0     0      0
## 3 0.006204757 0.3309204        0        0      0 0.9879      0     0      0
## 4 0.024096386 0.4236948        0        0      0 0.9922      0     0      0
##   CZlgpA CZvepA KmpA npscA
## 1      0 0.0105    0     0
## 2      0 0.0030    0     0
## 3      0 0.0121    0     0
## 4      0 0.0078    0     0
```

The exact calculation of attribute values for the total catchment of each site can take quite long (depending on the number of points): For each site, first the total catchment is delineated based on the DEM and then intersected with the map(s) provided. It must be decided on a case by case basis if the approximate calculation is good enough; often, the results of the two approaches are very similar (see comparison below).

Exact calculation:

```
# this might take a while
calc_attributes_sites_exact(sites_map = "sites",
                            input_raster = "landuse_r",
                            stat_rast = "percent",
                            attr_name_rast = "luseE",
                            input_vector = c("geology", "psources"),
                            stat_vect = c("percent", "count"),
                            attr_name_vect = c("GEO_NAME", "nps"),
                            round_dig = 4)
## Intersecting maps for 60 sites ...

## Joining new attributes to attribute table ...

##
## New attributes values are stored as 'H2OArea', 'luseE_1', 'luseE_2',
'luseE_3', 'luseE_4', 'luseE_5', 'luseE_6', 'luseE_7', 'CZfgp', 'CZbgp',
'CZamp', 'Kmp', 'CZgp', 'CZvep', 'CZigp', 'CZlgp', 'nps' in 'sites'.


sites <- readVECT("sites", ignore.stderr = TRUE)
head(sites@data, n = 4)
## cat site_id value str_edge dist NEAR_X NEAR_Y locID pid netID rid
## 1 1 1 1 65 85.04240 631046.1 226074.1 1 1 21 64
## 2 2 4 1 174 47.80371 632011.9 225175.7 2 2 21 171
## 3 3 5 1 67 29.83919 631203.4 224771.5 3 3 21 66
## 4 4 7 1 175 31.22663 631787.3 224883.8 4 4 21 172
## upDist distalong ratio H2OAreaA lusep_1A lusep_2A lusep_3A
## 1 2828.29 147.9922 0.1536049 0.61 0.3613933 0 0.00000000
## 2 1368.34 114.8528 0.7124438 2.06 0.1688907 0 0.05838198
## 3 2012.90 264.8528 0.4950953 0.73 0.5667011 0 0.09617373
## 4 1286.33 104.7310 0.6591677 1.28 0.4892905 0 0.06224900
## lusep_4A lusep_5A lusep_6A lusep_7A CZampA CZbgpA CZfgpA CZgpA CZigpA
## 1 0.071117562 0.5674891 0 0 0 0.9895 0 0 0
## 2 0.044203503 0.7285238 0 0 0 0.9970 0 0 0
```

```
## 3 0.006204757 0.3309204 0 0 0 0.9879 0 0 0
## 4 0.024096386 0.4236948 0 0 0 0.9922 0 0 0
## CZlgpA CZvepA KmpA npscA H2OArea luseE_1 luseE_2 luseE_3 luseE_4
## 1 0 0.0105 0 0 0.6201 0.3613933 0 0.00000000 0.071117562
## 2 0 0.0030 0 0 2.1105 0.1727079 0 0.05970149 0.045202559
## 3 0 0.0121 0 0 0.6696 0.6061828 0 0.07258065 0.008064516
## 4 0 0.0078 0 0 1.2726 0.5169731 0 0.06577086 0.025459689
## luseE_5 luseE_6 luseE_7 CZfgp CZbgp CZamp Kmp CZgp CZvep CZigp CZlgp nps
## 1 0.5674891 0 0 0 0.9895 0 0 0 0.0105 0 0 0
## 2 0.7223881 0 0 0 0.9969 0 0 0 0.0031 0 0 0
## 3 0.3131720 0 0 0 0.9843 0 0 0 0.0157 0 0 0
## 4 0.3917963 0 0 0 0.9917 0 0 0 0.0083 0 0 0
```

In both alternatives, the catchment area for each site is calculated automatically ('H2OAreaA' for `calc_attributes_sites_appox` and 'H2OArea' for `calc_attributes_sites_exact`). In most cases, the difference between the two alternative methods is small:

```
# plot comparison of approximate and exact calculation
par(bty = "l", las = 1)
plot(sites$lusep_1A, sites$luseE_1, ylim = c(0,1), xlim = c(0,1),
     pch = 19, col = "dodgerblue",
     ylab = "fraction land use type 1 exact calculation",
     xlab = "fraction land use type 1 approximate calculation")
abline(a=0, b=1, lty = 2)
```

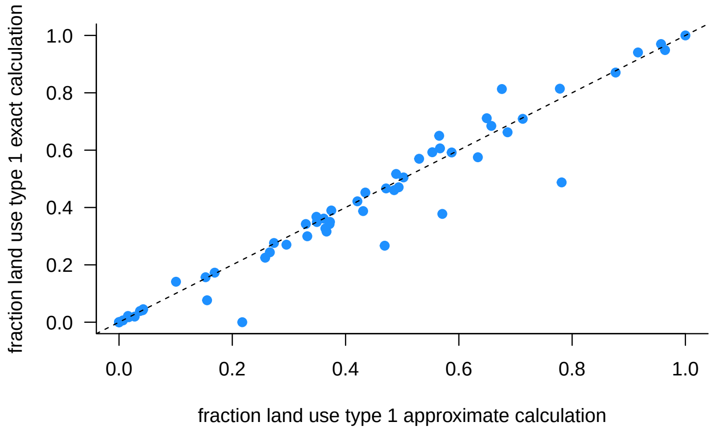

E - Export data

Merge measurements to sampling sites

The acutal measurements at the sampling sites may be stored in a separate file. This is particularly useful if there are measurements at sites at several dates. In this example, there are three measurement time points for each site. `measurements` can be given as a date.frame or data.table object, or as a file path to a table. In the latter case, the specifications of the file e.g. the character separating columns (`sep`) or the character used for decimal points (`dec`) must be specified. See `?read.table` for details.

```r
# create a copy of the sites file in case something goes wrong
execGRASS("g.copy",
          parameters = list(
            vector = "sites,sites_s"
          ))
## Copy vector <sites@PERMANENT> to current mapset as <sites_s>


# merge a table of measurements to the sampling sites based on the column 'site_id'
merge_sites_measurements(measurements = system.file(
                            "extdata", "nc", "obs_data.csv", package = "openSTARS"),
                         site_id = "site_id", sep = ",", dec = ".")

# note the dublicated rows, and the new columns at the end
sites <- readVECT("sites", ignore.stderr = TRUE)
head(sites@data, n = 6)
## cat site_id value str_edge dist NEAR_X NEAR_Y locID pid netID rid
## 1 1 1 1 65 85.04240 631046.1 226074.1 1 1 21 64
## 2 2 1 1 65 85.04240 631046.1 226074.1 1 2 21 64
## 3 3 1 1 65 85.04240 631046.1 226074.1 1 3 21 64
## 4 4 4 1 174 47.80371 632011.9 225175.7 2 4 21 171
## 5 5 4 1 174 47.80371 632011.9 225175.7 2 5 21 171
## 6 6 4 1 174 47.80371 632011.9 225175.7 2 6 21 171
## upDist distalong ratio H2OAreaA lusep_1A lusep_2A lusep_3A lusep_4A
## 1 2828.29 147.9922 0.1536049 0.61 0.3613933 0 0.00000000 0.07111756
## 2 2828.29 147.9922 0.1536049 0.61 0.3613933 0 0.00000000 0.07111756
## 3 2828.29 147.9922 0.1536049 0.61 0.3613933 0 0.00000000 0.07111756
## 4 1368.34 114.8528 0.7124438 2.06 0.1688907 0 0.05838198 0.04420350
## 5 1368.34 114.8528 0.7124438 2.06 0.1688907 0 0.05838198 0.04420350
## 6 1368.34 114.8528 0.7124438 2.06 0.1688907 0 0.05838198 0.04420350
## lusep_5A lusep_6A lusep_7A CZampA CZbgpA CZfgpA CZgpA CZigpA CZlgpA CZvepA
## 1 0.5674891 0 0 0 0.9895 0 0 0 0 0.0105
## 2 0.5674891 0 0 0 0.9895 0 0 0 0 0.0105
## 3 0.5674891 0 0 0 0.9895 0 0 0 0 0.0105
## 4 0.7285238 0 0 0 0.9970 0 0 0 0 0.0030
## 5 0.7285238 0 0 0 0.9970 0 0 0 0 0.0030
## 6 0.7285238 0 0 0 0.9970 0 0 0 0 0.0030
## KmpA npscA H2OArea luseE_1 luseE_2 luseE_3 luseE_4 luseE_5 luseE_6
## 1 0 0 0.6201 0.3613933 0 0.00000000 0.07111756 0.5674891 0
## 2 0 0 0.6201 0.3613933 0 0.00000000 0.07111756 0.5674891 0
## 3 0 0 0.6201 0.3613933 0 0.00000000 0.07111756 0.5674891 0
## 4 0 0 2.1105 0.1727079 0 0.05970149 0.04520256 0.7223881 0
## 5 0 0 2.1105 0.1727079 0 0.05970149 0.04520256 0.7223881 0
## 6 0 0 2.1105 0.1727079 0 0.05970149 0.04520256 0.7223881 0
## luseE_7 CZfgp CZbgp CZamp Kmp CZgp CZvep CZigp CZlgp nps X obs_time
## 1 0 0 0.9895 0 0 0 0.0105 0 0 0 1 2018-01-01
## 2 0 0 0.9895 0 0 0 0.0105 0 0 0 2 2018-03-01
## 3 0 0 0.9895 0 0 0 0.0105 0 0 0 3 2018-07-01
## 4 0 0 0.9969 0 0 0 0.0031 0 0 0 4 2018-01-01
## 5 0 0 0.9969 0 0 0 0.0031 0 0 0 5 2018-03-01
## 6 0 0 0.9969 0 0 0 0.0031 0 0 0 6 2018-07-01
## val1 val2
## 1 4.244630 13.752719
```

```
## 2 5.044714 13.312986
## 3 4.769702 10.704348
## 4 5.000448 9.416903
## 5 4.026259 13.037051
## 6 4.946206 14.210326
```

Write all files to an ssn folder

All files needed (edges, sites and optionally prediction sites) are written to the file path provided and can then be read in by the SSN package.

```
### Write all files to an ssn folder
ssn_dir <- file.path(getwd(), 'nc.ssn')
export_ssn(ssn_dir, predictions = "preds")
## Error in export_ssn(ssn_dir, predictions = "preds"): /home/mira/_Backup/05_Serior/14_Article_openSTAI
list.files(ssn_dir)
##  [1] "binaryID.db" "distance"    "edges.dbf"   "edges.prj"   "edges.shp"
##  [6] "edges.shx"   "netID12.dat" "netID21.dat" "netID44.dat" "netID53.dat"
## [11] "netID54.dat" "netID60.dat" "preds.dbf"   "preds.prj"   "preds.shp"
## [16] "preds.shx"   "sites.dbf"   "sites.prj"   "sites.shp"   "sites.shx"
```

Try with SSN package

Please note that this is for illustration only; the response variable `val1` contains random values. Therefore, the model fit bad and the model is not meaningful.

```
library(SSN)
## Loading required package: RSQLite

## Loading required package: sp

# import
ssn_obj <- importSSN(ssn_dir, o.write = TRUE)
plot(ssn_obj, 'val1')
```

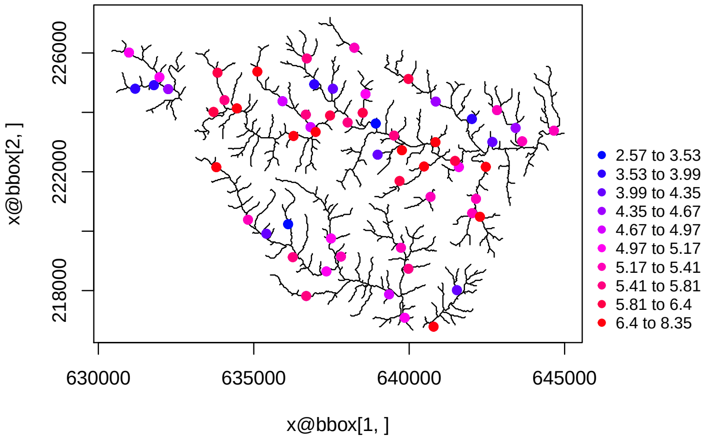

```r
# Create Distance Matrix
createDistMat(ssn_obj, o.write = TRUE)
dmats <- getStreamDistMat(ssn_obj)

ssn_obj.Torg <- Torgegram(ssn_obj, "val1", nlag = 20, maxlag = 15000)
plot(ssn_obj.Torg)
```

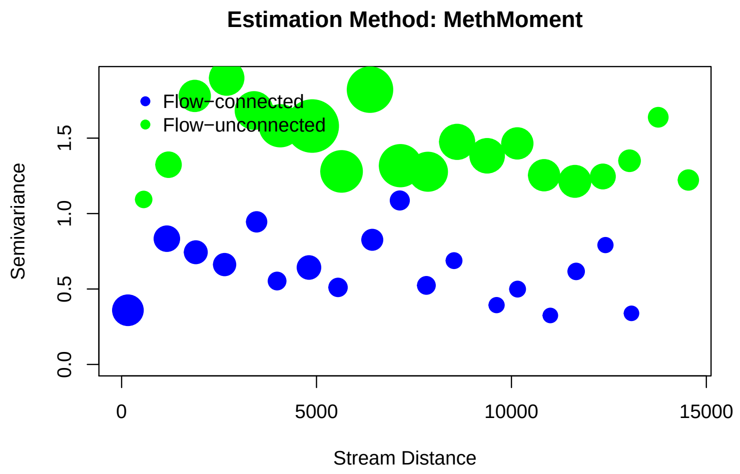


```r
names(ssn_obj@data)
##  [1] "cat"        "stream"     "flow_accu" "changed"   "netID"     "rid"
##  [7] "OBJECTID"   "Length"     "upDist"    "H2OArea"   "rcaArea"   "lusep_1_e"
## [13] "lusep_1_c" "lusep_2_e" "lusep_2_c" "lusep_3_e" "lusep_3_c" "lusep_4_e"
## [19] "lusep_4_c" "lusep_5_e" "lusep_5_c" "lusep_6_e" "lusep_6_c" "lusep_7_e"
## [25] "lusep_7_c" "CZamp_e"   "CZamp_c"   "CZbgp_e"   "CZbgp_c"   "CZfgp_e"
## [31] "CZfgp_c"   "CZgp_e"    "CZgp_c"    "CZigp_e"   "CZigp_c"   "CZlgp_e"
## [37] "CZlgp_c"   "CZvep_e"   "CZvep_c"   "Kmp_e"     "Kmp_c"     "npsc_e"
## [43] "npsc_c"

names(ssn_obj)
## $Obs
##  [1] "cat"       "site_id"   "value"     "str_edge"  "dist"      "NEAR_X"
##  [7] "NEAR_Y"    "locID"     "pid"       "netID"     "rid"       "upDist"
## [13] "distalong" "ratio"     "H2OAreaA"  "lusep_1A"  "lusep_2A"  "lusep_3A"
## [19] "lusep_4A"  "lusep_5A"  "lusep_6A"  "lusep_7A"  "CZampA"    "CZbgpA"
## [25] "CZfgpA"    "CZgpA"     "CZigpA"    "CZlgpA"    "CZvepA"    "KmpA"
## [31] "npscA"     "H2OArea"   "luseE_1"   "luseE_2"   "luseE_3"   "luseE_4"
## [37] "luseE_5"   "luseE_6"   "luseE_7"   "CZfgp"     "CZbgp"     "CZamp"
## [43] "Kmp"       "CZgp"      "CZvep"     "CZigp"     "CZlgp"     "nps"
## [49] "X"         "obs_time"  "val1"      "val2"

ssn_obj <- additive.function(ssn_obj, "H2OArea", "computed.afv")

# non-spatial model
ssn_obj.glmssn0 <- glmssn(val1 ~ upDist, ssn.object = ssn_obj,
                          CorModels = NULL)
summary(ssn_obj.glmssn0)
```

```
##
## Call:
## glmssn(formula = val1 ~ upDist, ssn.object = ssn_obj, CorModels = NULL)
##
## Residuals:
##      Min       1Q   Median       3Q      Max
## -2.46856 -0.70208  0.08094  0.63487  3.51566
##
## Coefficients:
##              Estimate Std. Error t value Pr(>|t|)
## (Intercept) 4.544e+00  1.477e-01  30.757  < 2e-16 ***
## upDist      5.503e-05  1.716e-05   3.207  0.00159 **
## ---
## Signif. codes:  0 '***' 0.001 '**' 0.01 '*' 0.05 '.' 0.1 ' ' 1
##
## Covariance Parameters:
##  Covariance.Model Parameter Estimate
##            Nugget   parsill     1.17
##
## Residual standard error: 1.080606
## Generalized R-squared: 0.05462368

# same as
summary(lm(val1 ~ upDist, getSSNdata.frame(ssn_obj)))
##
## Call:
## lm(formula = val1 ~ upDist, data = getSSNdata.frame(ssn_obj))
##
## Residuals:
##     Min      1Q  Median      3Q     Max
## -2.4686 -0.7021  0.0809  0.6349  3.5157
##
## Coefficients:
##              Estimate Std. Error t value Pr(>|t|)
## (Intercept) 4.544e+00  1.477e-01  30.757  < 2e-16 ***
## upDist      5.503e-05  1.716e-05   3.207  0.00159 **
## ---
## Signif. codes:  0 '***' 0.001 '**' 0.01 '*' 0.05 '.' 0.1 ' ' 1
##
## Residual standard error: 1.081 on 178 degrees of freedom
## Multiple R-squared:  0.05462,    Adjusted R-squared:  0.04931
## F-statistic: 10.28 on 1 and 178 DF,  p-value: 0.00159


# with random effect to account for multiple measurements at the same site
ssn_obj.glmssn0.mixed <- glmssn(val1 ~ upDist, ssn.object = ssn_obj,
                          CorModels = c("locID"))
summary(ssn_obj.glmssn0.mixed)
##
## Call:
## glmssn(formula = val1 ~ upDist, ssn.object = ssn_obj, CorModels = c("locID"))
##
## Residuals:
```

```
##      Min       1Q   Median       3Q      Max
## -2.46856 -0.70208  0.08094  0.63487  3.51566
##
## Coefficients:
##              Estimate Std. Error t value Pr(>|t|)
## (Intercept) 4.544e+00  2.376e-01  19.128   <2e-16 ***
## upDist      5.503e-05  2.759e-05   1.994   0.0476 *
## ---
## Signif. codes:  0 '***' 0.001 '**' 0.01 '*' 0.05 '.' 0.1 ' ' 1
##
## Covariance Parameters:
##  Covariance.Model Parameter Estimate
##             locID   parsill    0.915
##            Nugget   parsill    0.273
##
## Residual standard error: 1.090085
## Generalized R-squared: 0.02185935
```

```
# spatial model
ssn_obj.glmssn1 <- glmssn(val1 ~ upDist , ssn.object = ssn_obj,
                          CorModels = c("Exponential.taildown", "Exponential.tailup"),
                          addfunccol = "computed.afv")
summary(ssn_obj.glmssn1)
##
## Call:
## glmssn(formula = val1 ~ upDist, ssn.object = ssn_obj, CorModels =
c("Exponential.taildown",
## "Exponential.tailup"), addfunccol = "computed.afv")
##
## Residuals:
## Min 1Q Median 3Q Max
## -2.3282 -0.6494 0.1794 0.7166 3.5716
##
## Coefficients:
## Estimate Std. Error t value Pr(>|t|)
## (Intercept) 4.605e+00 2.824e-01 16.308 <2e-16 ***
## upDist 3.303e-05 3.263e-05 1.012 0.313
## ---
## Signif. codes: 0 '***' 0.001 '**' 0.01 '*' 0.05 '.' 0.1 ' ' 1
##
## Covariance Parameters:
## Covariance.Model Parameter Estimate
## Exponential.tailup parsill 0.708
## Exponential.tailup range 70120.231
## Exponential.taildown parsill 0.274
## Exponential.taildown range 335.318
## Nugget parsill 0.279
##
## Residual standard error: 1.122529
## Generalized R-squared: 0.005914411
```

```
varcomp(ssn_obj.glmssn1)
##                VarComp  Proportion
## 1    Covariates (R-sq) 0.005914411
## 2   Exponential.tailup 0.558247264
## 3 Exponential.taildown 0.216000784
## 4               Nugget 0.219837542
```
