# Supplementary material for: Preparing GIS data for analysis of stream monitoring data: The R package openSTARS
Source: PLoS One. 2020 Sep 17;15(9):e0239237. doi: 10.1371/journal.pone.0239237 (PMC7498020; doi:10.1371/journal.pone.0239237)
Supplement: Attachment — Submitted filename: Response_to_Reviewers.pdf [file pone.0239237.s003.pdf]

Detailed reviewer response; answers are marked in green:

5. Review Comments to the Author

Reviewer #1: The submitted text "Preparing GIS data for analysis of stream monitoring data: The R package openSTARS" by Mira Kattwinkel at.al. describes a complete working licenses free library named "openStars" for R statistical environment. The software is dedicated on pre-processing of stream monitoring data for further statistical analysis, mainly by SSN. The tool is already available online and free for use. There is a manual which is easy to understand and reproduce.

We thank the reviewer for this positive general appraisal. We address all comments and problems in the following.

However, I didn't succeeded to reproduce provided example in Supporting_Information_S2. There are computational error with derive_streams(accum_threshold = 100, condition = T, clean = TRUE, burn = 10) when accum_threshold is equal to 100, failing to execute correctly calc_edges() and calc_sites(), mainly producing missing values for locID, pid and netID. The error does not exist for accum_threshold bigger than 170, but the results are different than those in supporting information. The used version of GRASS is 7.6 on R version 3.6.3 for Fedora 31.

We updated several parts of the code, including a further check in calc_edges() for complex confluences. It seems, that the correction of those was the problem here (because when accum_threshold is >= 170 there will be no complex confluences). The deviation in results for a different threshold, however, is correct because a different network is created.

We checked the example code using the updated package on Linux Mint 19, R 4.02, GRASS 7.8.3; Linux Mint 19, R 3.6.3, GRASS 7.4; Windows 10, R 4.0 and GRASS 7.6 and it run without errors. In case the reviewer still experiences computational errors, we would be very grateful for more information to be able to identify the problem (e.g. via https://github.com/MiKatt/openSTARS).

2.The text have to be revisited because the existence of non-clear statements, such as at p.8, line 170 where the following text exists "openSTARS and STARS yielded very similar results (2)", where reference to some index (2) is confusing.

We are sorry for the confusion. Apparently, during the upload process the “Fig” went missing. So (2) refers to Figure 2, which gives a map comparing streams and site locations derived with the two tools. We corrected this in the revised version and checked carefully for all other cross-references.

Another example is the sentence on p. 9, line 182, starting with "The major conceptional difference..." where it is claimed "that the STARS derives the stream network from a DEM, whereas openSTARS relies on an existing stream network in vector format", which is opposite to the previous text.

Thank you for spotting this typo. Of course, it should be the other way around and was corrected.

3.According the statistical part in Paragraph 3, I think that is too brief and incomplete. Secondly, it is not clear a correlation of what and how is computed. I only could guess that outliers are removed from computation, because it is very suspicious to have r bigger that 0.98 with so large outliers. Moreover, the correlation is not suitable because samples in poor agreement may have high

correlation. Likewise, the study of possible bias is also missing. As an option for revision the authors could consider any parametric or non-parametric test or at least a graphical tools, such as widely used in medicine Bland-Altman plots.

Unfortunately, also here the reference to Figure 3 was corrupted and we corrected it. Furthermore, we complemented the text with some more details for clarity. Additionally, we included a Bland-Altman plot as suggested by the reviewer in the Supporting Information for another way to compare the catchment attributes calculated with the two different tools. The same outliers can be identified in that plot and are discussed in detail in the Supporting Information.

Reviewer #2: The submission itself is more or less ready to go, and explains workflow variants, and in the supplementary materials also analysis in the SSD package. The points that I feel deserve attention are that GRASS is now 7.8.3, R 4.0.2, and some of the package code is showing its age. For example, check_compl_confluences() now reports: "The command: v.to.db --quiet map=streams_v option=length type=line columns=length_new produced an error (1) during execution: ERROR: Column

A possible vulnerability is that both GRASS and R (sp and sf packages) have adapted to changes in PROJ and GDAL, so that the handling of coordinate reference systems across the boundary between the two interfaces software systems needs checking, and relying on PROJ strings may not be as reliable as it has. So when checking the software and provided scripts for changes from version changes in R and GRASS, it would be very sensible to check whether any vulnerabilities are present as R moves from Proj4 to WKT2 string representations.

Thanks for this positive review and for rising the version issues.

We updated the package and examples to GRASS 7.8.3 and R 4.0.2. We are aware that a lot is going on regarding PROJ and GDAL. It seems that the transition in other R packages is still ongoing and there is currently no way to be sure what implementation will be stable. Therefore, we decided to carefully check that our current implementation is still working and will update the code accordingly once specific changes in other packaged we depend on are in place.

Particularly, we kept calc_sites(), the only function using sp::proj4string(x), as it is. The alternative to use GRASS v.db.select and v.in.ascii seems to be unsuitable for our purpose, because coordinates can only be imported as integers resulting in small derivations from point positions and stream segments. We did update the function setup_grass_environment(), however, so that it now uses the SG argument of initGRASS to setup the region of the location instead of taking the definitions manually from the dem.

Furthermore, we updated our code where v.to.db is used because its implementation was changed in GRASS 7.8 resulting in an error in our previous implementation. The newer version can create new columns but needs an overwrite flag if the column already exists, while the older versions needed the columns to be present before populating it.

To prevent confusing warning messages related to rgdal and proj4 in the plotting sections of the example workflow, we suppress warnings in that sections and inform the user about this for clarity.
